# Resilience, Emotional Intelligence, and Occupational Performance in Family Members Who Are the Caretakers of Patients with Dementia in Spain: A Cross-Sectional, Analytical, and Descriptive Study

**DOI:** 10.3390/jcm10184262

**Published:** 2021-09-20

**Authors:** María Nieves Gómez-Trinidad, Carlos Alexis Chimpén-López, Laura Rodríguez-Santos, Manuel Alfredo Moral, Juan Rodríguez-Mansilla

**Affiliations:** 1 Department of Medical-Surgical Therapy, Faculty of Nursing and Occupational Therapy, University of Extremadura, 10003 Cáceres, Spain; nievesgomez@unex.es; 2 Department of Medical-Surgical Therapy, Psychiatry Area, Faculty of Nursing and Occupational Therapy, University of Extremadura, 10003 Cáceres, Spain; 3 Department of Medical-Surgical Therapy, Psychiatry Area, Faculty of Medicine and Health Sciences, University of Extremadura, 06006 Badajoz, Spain; laura@unex.es; 4 Department of Religion, Oakwood University, Huntsville, AL 35896, USA; manuelalfredomoral@gmail.com; 5 ADOLOR Research Group, Department of Medical-Surgical Therapy, Faculty of Medicine and Health Sciences, University of Extremadura, 06006 Badajoz, Spain; jrodman@unex.es

**Keywords:** caretakers, resilience, emotional intelligence, dementia

## Abstract

Background: The concern in the scientific community for the study of people with dementia and their families is comprehensible, especially the importance of knowing the effects that caring for the patient has on their family dynamic, paying special attention to the main caregiver. The objective of this study was to analyze the relationship of resilience and emotional intelligence with functional performance in the main caregivers of people with dementia in Spain according to the phase of the disease. Methods: A cross-sectional, descriptive, and analytical study was carried out. A total of 144 primary family caregivers of patients with dementia in Spain were included in the study. The following variables were measured: sociodemographic, psychosocial, and occupational, as well as resilience and emotional intelligence. Results: The caregivers obtained a low moderate resilience (mean = 64.01 ± 14.5), an emotional intelligence bordering between moderate and high (mean = 78.48 ± 14.82), and a 61.8% self-care categorized as somewhat and quite a bit. The presence of higher levels of resilience in family caregivers of people with dementia were positively related to the time spent on self-care (r = 0.227; *p* = 0.033) and leisure (r = 0.262; *p* = 0.014), especially in the moderate phase of the disease, while in the severe phase, this relationship appeared with productivity (r = 0.355; *p* = 0.034). The higher levels of emotional intelligence were positively related to a greater time dedicated to self-care (r = 0.233, *p* = 0.005), as well as the data observed in the moderate and severe phase (r = 0.214; *p* = 0.046 and r = 0.398; *p* = 0.016 respectively). Conclusions: The primary caregivers of relatives with dementia who have higher levels of resilience and emotional intelligence spend more time on self-care and leisure activities, especially in the moderate phase of the disease.

## 1. Introduction

The impact of dementia goes far beyond individual effects at the cognitive, behavioral, physical, and functional level of the person who suffers from it. There are repercussions of the diseases in the family and in the social and economic environment [1]. Research indicates that the family is the main provider of care [2,3]. As such, it is imperative to understand the structural changes to which they are subjected given the existing responsibilities that are carried out under the act of caring. In this regard, we can define the main caregiver as “that person who cares for the patient in a manner that is seeking to meet physical, psychological, affective, social and functional needs” [4,5,6].

The overload that may affect the main caregiver is motivated by the presence of certain primary stressors (consequences of the deterioration of the patient and the care they require) and secondary (derived from the primary ones), causing symptoms related to changes in health, work, economy, and free time [6] that translate into difficulties for physical, psychosocial, and family functioning [7,8,9]. The care has to be adapted according to the stages of the dementia. In the initial or mild stage, there is an intellectual deterioration resulting in problems in the family, social, and occupational functioning, alteration of the wake–sleep rhythm, as well as disorientation in time, and problems in calculation and spelling that did not exist previously. The moderate stage is characterized by disorientation in time, space, and known places, behavioral, personality, and motor disturbances, clumsiness or restlessness, worsening memory problems, visual hallucinations, and intensified delusions. In the advanced or severe stage, cognitive and individual losses occur, presenting what is called “psychic death”, which precedes physical death [10,11].

The scientific literature that deals with family caregivers of people with dementia has had an enormous interest in the study of the impact of time and occupations in the main caregiver. In this line, studies, such as the one by González Galvez et al. [12], carried out in 2014, aimed at knowing the coping process in primary and informal caregivers of people with severe dependence. These studies reflect on how the loss of autonomy within the repercussions of the primary caregiver, as well as the shortage of time for themselves, combined with the pressures for the various roles to play, translate into an emotional impact and loss of freedom. Other research highlights that 33% of the main caregivers of Alzheimer’s patients abandon their personal care and food, clothing, and hairdressing care, among others [13]. Moreover, 59.8% of the main caregivers report not having time to dedicate “to themselves”, due to the permanent dedication that the care of the sick family member requires [14,15]. They also describe a break with the usual routine, loss of social relations, loss of dedication to hobbies, as well as a drop in income due to having to reduce or even abandon their work [7,16,17]. Research shows the frequency with which caregivers experience problems of anxiety, depression, and insomnia [10,18,19]. In addition, some studies indicate that sleep debt is known to have cumulative associations with the physical, mental, and cognitive health of caregivers [20].

On that basis, there is a need to know if the caregiver of a patient with dementia knows how to adaptively face conflicts while gaining something positive from the experience (learning or personal growth) [21], which is known as resilience. It is also interesting to know if the caregiver is an emotionally intelligent person, with a positive attitude, a motivation to try to achieve certain ends or purposes (self-motivation), control impulses (analyzing and recognizing emotions, as well as acting accordingly), tolerate frustration without giving up easily, and relate to others with empathy and assertiveness [22,23,24,25]. With both concepts, we move away from the traditional pathological approach to human behavior in adverse situations towards adaptive coping and its positive consequences for the primary caregiver to maintain a “balance” between different occupations: activities of daily living (ADL) instrumental activities of daily living (IADL), health management, rest and sleep, education, work, play, leisure, and social participation [26].

In this joint study of emotional intelligence and resilience, it is interesting to see how a relationship between the two qualities is established and how we can understand the former (emotional intelligence) as a “prerequisite” or “predictor” of the latter (resilience). Thus, the interest in expanding research on the relationship and proximity of both concepts is further deepened [27,28]. In 2011, Colop [29] carried out a study on young adult with the aim of determining the relationship between these variables, and concluded that emotional intelligence is not essential for the existence of resilience in a person. The study showed that 90% of the participants had an adequate level of resilience while emotional intelligence appeared in a lower percentage, possibly due to other factors not identified in the research.

Other recent studies demonstrate the interest of investigating resilience in other populations, such as women with breast cancer or cocaine use [30,31]. Studies focusing on the resilience of caregivers of patients with advanced or chronic illness demonstrate its influence on adaptive coping, as well as reduced perceptions of burden and stress. [32]. With regard to resilience in caregivers of people with Alzheimer’s [33], high levels of resilience and fewer somatic and depression symptoms have been observed in caregivers who have a moderate to heavy burden on the care of these patients. Emotional intelligence in the caregivers of people with dementia has been little studied, so there is a strong research interest on this variable. Studies have focused more on research in the field of employment, education, or even health, but with few samples similar to ours. However, some works, such as those of Vázquez et al. [34] have already shown the link between lower levels of emotional intelligence and greater depressive symptoms in caregivers of long-term illnesses such as Alzheimer’s disease. Similar conclusions are drawn from different studies [35,36] relating, in an inversely proportional way, the levels of emotional intelligence with the perception of burden, anxiety, and depression. However, the reviewed research has not addressed the relationship between resilience and emotional intelligence in the practical and functional coping with a stressful situation, such as having a family member with dementia. Therefore, the objective of this study is to analyze the relationship of resilience and emotional intelligence with functional performance in the main caregivers of people with dementia in Spain according to the severity of the disease.

## 2. Materials and Methods

### 2.1. Design

This was a cross-sectional, descriptive, and analytical study. 

### 2.2. Ethical Aspects

Ethical approval was received from the Bioethical Commission of the University of Extremadura in Spain (Registration number: 32/2016). Written informed consent was provided by all the participants.

### 2.3. Participants

The study population consisted of 388 primary family caregivers belonging to 13 Associations of Relatives of Dementia in the Extremadura region (Spain).

The study sample consisted of 144 family members. The main caregivers of family members with dementia, and those who met the following inclusion criteria were included in the study: 18 years of age or older, the main caregiver and relative of the patient, and willing to participate in the study and provide informed consent.

### 2.4. Procedure

Meetings were arranged with each of the collaborating associations, which made it possible to publicize the objective of the study and resolve any doubts. There were documents that the main family members/caregivers had to fill out. From each different association, a reference professional was assigned outside the study (social worker or psychologist) who oversaw distributing and collecting the different documents from the relatives.

Once filled out, the participant inserted the documents in an envelope, closed it and handed it over to the reference professional of each association. The collection time was marked as one month from the delivery of the documentation. The collected documents were then encrypted with keys, archived, and guarded. Questionnaires that were not duly completed were excluded.

### 2.5. Study Variables and Data Collection Instruments

Sociodemographic, psychosocial, and occupational variables: Our questionnaire was developed to collect the sociodemographic, psychosocial, and occupational variables in the study population. The questions were directed to whichever family member was the main caregiver of the patient.

1. Sociodemographic variables: sex (man, woman), age, number of children, number of people living at home, marital status (single, married, separated with or without a partner, widowed), educational level (no education, literate, basic, baccalaureate or vocational training, university studies), work outside the home or paid (full/part time, retired-pensioner, unemployed, exclusivity in housework, other).
2. Psychosocial variables: relationship with the sick relative (mother, father, others), other people in charge of the caregiver, number of people that the caregiver has under his/her care, age of the patient, hours a day and days a week that they dedicate care, time spent in the situation of caring for the patient, presence of formal support (day center, home help, residence, economic benefit) and/or informal (family, friends, others), knowledge about the family member’s illness and need for more training on it. Living out the experience: whether or not there is a positive perception about living out the experience and feelings that this generates (positive response: feelings of usefulness, love, dedication, gratitude towards the patient, feeling of personal growth, or others. Negative response: overload, physical health problems, mental health problems, others).
3. Occupational performance: for the division of occupational performance areas, those based on the Canadian Model of Occupational Performance (C.M.O.P.) [37] were used, classified as Self-Care, Productivity, and Leisure. Each of these areas were evaluated using a Likert-type scale where caregivers responded based on the time spent in each area (1 = not at all, 2 = little, 3 = somewhat, 4 = quite a bit, 5 = a lot). The caregivers were also asked to assess the satisfaction they felt in relation to the time they dedicated by means of a dichotomous answer of yes or no. A sleep assessment was also included, in which the caregivers were asked about the number of hours devoted to sleep per day and for the consideration of having a restful sleep.

### 2.6. Clinical and Psychological Variables

1. Diagnosis: Alzheimer’s disease, Vascular Dementia, Lewy body dementia, Frontotemporal dementia, and others, along with the phase of the disease (mild, moderate, and severe). This diagnosis was given by a medical specialist.
2. Resilience: the Connor–Davidson Resilience Scale 25 eur (CD-RISC 25eur) was used; official European version translated into Spanish, derived from the original Connor–Davidson Resilience Scale [38]. It was directly purchased from the authors upon request for permission for its use, together with the unpublished manual of the scale [39], revised on 11 January 2015. It is the resilience scale most commonly used [40] in different studies in the same study population, which confirms its validity and reliability [40,41]. It is a tool made up of 25 items that, based on Richardson’s theoretical model [42], comprises five components considered necessary in the assessment of resilience: personal competence, confidence in one’s own intuition and tolerance to adversity, positive acceptance of change, control, and spirituality. The data provided should respond to a situation perceived during the last month, not to a situation experienced at a specific moment. The results fluctuate between 0 and 100; the higher the score, the higher the level or capacity of resilience. Considering the values provided by previous studies [33,40], the cut-off point was established at 74 (coinciding with the fourth quartile) to distinguish the two groups of caregivers: caregivers with high resilience and caregivers with moderate or low resilience.
3. Emotional intelligence: the TMMS-24 Emotional Intelligence scale was used, a version adapted to Spanish in 2004 by Fernández et al. [43], from the Trait Meta-Knowledge Scale on Emotional States, Trait Meta-Mood Scale [35], elaborated by the research group of Salovey and Mayer in 1995. It consists of a self-report that evaluates the perception of intrapersonal emotional intelligence. It consists of 24 items distributed in three subscales, Emotional Attention, Emotional Clarity, and Emotional Repair, with eight items each. The cut-off point was established at 79, coinciding with the median, dividing the sample into individuals with high emotional intelligence and individuals with low emotional intelligence.

### 2.7. Statistical Analysis

A descriptive analysis of all the variables was performed, calculating distribution measures (frequencies and percentages) in the case of qualitative variables and measures of centralization (means) and dispersion (standard deviation, maximum, and minimum) for continuous quantitative variables. The normality of continuous quantitative variables was studied through the Kolmogorov–Smirnov test, and we made the decision to carry out non-parametric tests. For the comparison of the mean scores of the scales between different groups, the Kruskal–Wallis H was used (post hoc: Dwass–Steel–Critchlow-Fligner pairwise comparison) and to analyze the relationships between the variables, bivariate analyses were carried out using the Spearman correlation coefficient.

The main relationships between the categorial variables were analyzed byχ^2^ test, followed by the Z test with Bonferroni’s correction to identify the categories involved in the correlations.

The analyses and graphical representations were carried out in IBM SPSS Statistics 21 and Jamovi 1.2. The observed differences were considered to be statistically significant for values of *p* ≤ 0.05.

## 3. Results

Of the 388 questionnaires initially delivered, 167 were returned. After reviewing them for correct completion and considering the study selection criteria, a total sample of 144 participants was reached, with 23 questionnaires being eliminated due to non-compliance with the criteria inclusion, specifically, not being the main caregiver.

**Figure 1:**
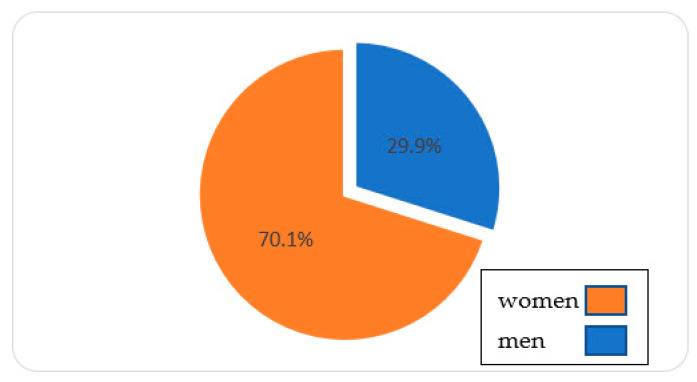
Sociodemographic: Sex.

**Figure 2:**
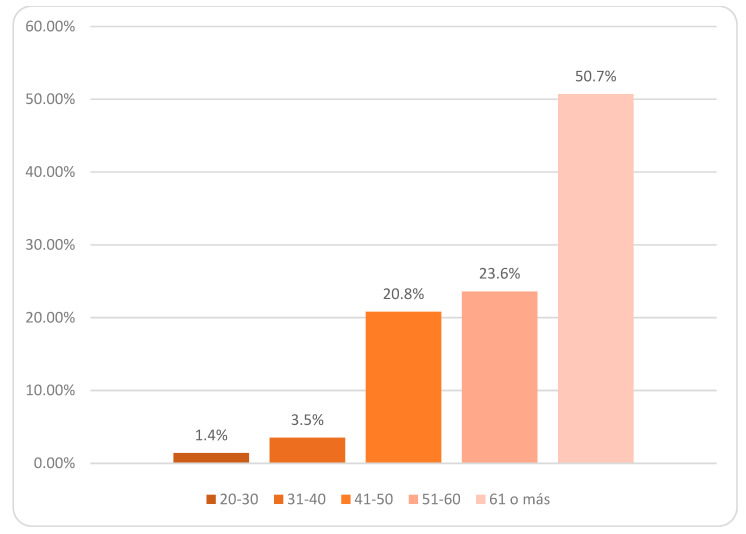
Sociodemographic: Age.

**Figure 3:**
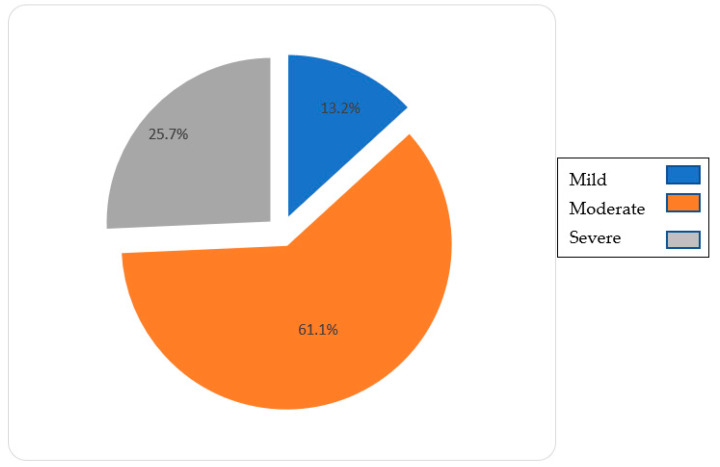
Dementia phase.

Regarding sociodemographic variables, the majority (70.1%) of the caregivers were women (Figure 1). Most of the caregivers were aged 40 and up, making up 95% of the total sample (Figure 2). Among the dementia patients, 66.7% had Alzheimer’s disease. The 61.1% were in a moderate phase of the disease (Figure 3). The rest of the sociodemographic, psychosocial, and clinical variables are described in Supplemental Tables S1 and S2. Table 1 shows the most significant socio-demographic variables according to the stages of severity.

### 3.1. Occupational Performance

The caregivers were asked to express their satisfaction with their occupational performance. When it came to time dedicated to self-care, 61.1% of the caregivers reported that they were satisfied with it. Additionally, 65.3% of the caregivers responded that they were satisfied with the time they dedicated to productivity. However, only 32.6% were satisfied with the time they spent doing leisurely activities. The surveyed caregivers slept an average of 6.39 ± 36 h a day, with 59.4% reporting that they did not experience a restful sleep (Table 2). No significant differences in occupational aspects were found when analyzed by disease stage.

### 3.2. Resilience and Emotional Intelligence

Table 3 presents the mean scores obtained in the scales of the global sample, according to the phase in which they are found: mild, moderate, and severe.

The caregivers obtained a mean score of 64.01 ± 14.5 on the resilience scale (moderate-low resilience) and a mean of 78.48 ± 14.82 on the emotional intelligence scale (emotional intelligence on the borderline towards high).

The results show the highest average levels of emotional intelligence and resilience in the mild phase of the disease.

Occupational performance presented similar means in the different phases of the disease, except in leisure, which was scored at a lower level for caregiving during the severe phase of the disease.

### 3.3. Resilience and Occupational Performance

In each of the analyses carried out, the global sample and the divided sample were differentiated according to the phases of dementia (mild, moderate, and severe) (Table 4).

Global sample: direct correlations were observed between the time spent caring for oneself and leisure and resilience, in that a longer time spent on self-care (r = 0.196; *p* = 0.019) and leisure led to a higher level of resilience (r = 0.172; *p* = 0.040). The time dedicated to productivity was not related to the level of resilience (r = 0.091; *p* = 0.278).

Mild phase dementia: the previously mentioned correlations were not observed within the group of caregivers of patients in a mild phase of dementia, and no correlation was statistically significant.

Dementia in moderate phase: in the case of caregivers of patients with dementia in the moderate phase, the same direct correlations were observed as in the global sample, between the level of resilience and the time dedicated to self-care (r = 0.227; *p* = 0.033) and leisure (r = 0.262; *p* = 0.014).

Dementia in severe phase: in caregivers of patients with dementia in the severe phase, the time dedicated to productivity increased as the level of resilience increased (r = 0.355; *p* = 0.034).

### 3.4. Emotional Intelligence and Occupational Performance

Global sample: regarding the relationship between occupational performance and emotional intelligence in general (without specifying in each scale), only the time dedicated to self-care appeared to be related to emotional intelligence, such that, with higher emotional intelligence came more time dedicated to self-care (r = 0.233; *p* = 0.005) (Table 5). This relationship also appeared in the moderate and severe phase, but not in the mild phase.

When particularizing in each subscale of emotional intelligence (Table 5), it was observed that the time dedicated to self-care was mainly related to better levels of repair; although this relationship did not become statistically significant, a clear trend was observed (*p* = 0.075). On the other hand, the more time was dedicated to leisure, the higher the level of repair that was observed (*p* = 0.019).

In the case of caregivers of patients with moderate and severe dementia, we observed that the higher the level of emotional intelligence, the more time spent on self-care (r = 0.21; *p* = 0.046; r = 0.398; *p* = 0.016 respectively), without any other correlation being significant.

When particularizing by subscales and after post hoc tests (Table 6), we observed in the caregivers of patients with dementia that the time spent on leisure was much higher in people with excellent repair compared to those who should improve their repair (W = 3.983; *p* = 0.013). In the moderate phase, the time spent on self-care was longer in people with adequate repair and lower in people who should improve their repair (W = 3.695; *p* = 0.024). However, in the severe phase, the time spent on leisure was much higher in people with excellent repair compared to the rest (excellent repair vs. should improve the repair: W = 4.089; *p* = 0.011; excellent repair vs. adequate repair: W = 3.798; *p* = 0.02). 

## 4. Discussion

Scientific evidence [33,40,41,42,43,44,45,46,47,48,49,50,51] has demonstrated the need to determine the “predictors” or “favoring” factors of resilience, specifying characteristics such as being a relative of the patient, the least degree of cognitive deterioration, the presence of social and family support, faith, humor, positivity and optimism, adequate information about the disease, and feelings of closeness and love towards the sick relative as the factors that are repeated the most in previous investigations. The consequences of this capacity or personality trait in the primary caregiver are less specific.

Some studies [33,41,46,52,53] agree about the positive correlation between resilience and fewer mental (depression) and physical health problems, as well as a lower risk of institutionalization of the patient. High levels of resilience appear to be related to personal growth [31], with the implementation of skills and competencies of work commitment [49,52,54,55], as well as with a greater capacity for self-care [41,52] and the more benign perception of aspects derived from care [47]. These consequences are the keys that can help us understand our results, since the presence of higher levels of resilience favors control and mastery of the situation, self-efficacy, and, therefore, the ingenuity to implement the necessary coping strategies and skills. These would include taking care of oneself, coinciding with the study by Ross et al. [54], where it is concluded that resilient caregivers recognize that if they do not make their health a priority, they are also exposed to physical and mental problems that make caring for their sick relative difficult.

Without specifying the type of self-care to which they refer, Crespo and Fernández [41,52] anticipate the influence of resilience as a protector of self-care, referring, in general terms, to health care, such as a decreased use of psychotropic drugs. Our work goes a little further to investigate the consequences of a resilient personality and its relationship with a better management of self-care in caregivers, referring to basic activities such as feeding, cleaning and dressing. This agrees with the results of the 2014 research by Bull [49], which pointed to the relationship between high levels of resilience and the use of strategies to manage time and take care of oneself. This included things like an adequate diet and using support networks such as home service so that caregivers can dedicate more time to housework, their career, and their social life. On the other hand, the conclusions of our work reveal differences according to the clinical phase of the disease, so the influence of resilience will be modified. In this regard, in the severe phase of the disease, we find statistical significance in the relationship between resilience and productivity (something that does not appear in the general sample or in any other phase). The explanation for this can be found in the clinical course of dementia. The severe phase is characterized by a loss of cognitive, intellectual, physical, and functional capacities, reaching a state of “psychic death” that precedes the physical one [10]. In this period, the type of care that the patient requires can be partially delegated to an external resource or support, allowing the main caregiver to breathe in a more relaxed or less stressful way compared to previous phases. It could be that the caregiver has already assumed the mental exhaustion of the illness condition, developed coping skills, and has come to understand the disease and its resulting behavioral alterations in the patient. This is where they “force” the presence of a reference figure, easily recognizable to the person with dementia, thereby helping to reduce the alterations produced in daily life.

In this study, the main caregivers, speak about having to be the person who carries the burden of care in the initial and moderate phases, since the presence of delusions and hallucinations pushes them to delegate care or makes them feel “less comfortable” in the relay. Garre-Olmo et al. [56] agree on this approach by expressing the difficulty generated by the patient’s mood and behavior disorders, which become a source of burden for the main caregiver, especially in the initial phase and intermediate disease. In the same way, Losada et al. [57] described the burdens faced by the family caregiver in the first phases of the disease, where they much face all the challenges it presents and dedicate themselves wholly to the patient and abandon their usual activities. In the severe phase, this “overload” disappears due to the clinical evolution of the patient, reaching a state of deterioration that is limited to basic care, together with the affection shown by the family member, which the patient will never fail to recognize [58]. At this time, the caregiver considers taking up activities, at least the main ones that they had to abandon, which can largely explain the results found in our work.

The study of emotional intelligence in our population of interest has stood out for revealing the negative consequences related to low levels of emotional intelligence. Along these lines, two scientific investigations [34,35] stand out that both relate low levels of emotional intelligence with depressive symptoms and a greater perception of burden and anxiety. However, Vázquez et al. [34] express the inability to attribute the cause or consequence of depressive symptoms to low emotional intelligence, pending further studies. Later, Gazquez et al. [59] concluded positive effects when determining the influence between skills related to emotional intelligence and a lower perception of stress and overload in the caregiver. In the same line of positive approach, Zijlmans et al. [54] and Sitges and Bonete [28] speak of emotional intelligence training as an adapted strategy for coping, for either formal or informal caregivers. Their conclusions reveal the capacity of emotional intelligence to avoid the worsening of negative emotions (e.g., anxiety or discomfort) or even to favor the management of such emotions, through different strategies. This last data, revealed by Zijlmans et al. [60], together with the research work of Portal [61] in 2014, concludes a relationship between emotional intelligence and a greater perception of control of the situation and coping by the caregiver, which allows us to reflect on the results found in our study. The highest levels of emotional intelligence, mainly in terms of clarity and emotional repair, were related to a better management of the time dedicated to self-care and leisure of the main caregiver. We confirm the positive effect of emotional intelligence on adapted coping; in this case, we are talking about practical areas such as the occupational performance of the caregiver himself. At the same time, we recognize that having resilience [47] and high emotional intelligence can foster a less harmful or toxic view of the situation [60] and help caregivers manage their emotions.

A detail that invites us to reflect on this data is the presence of high values in “emotional repair”. Within the components of emotional intelligence, repair is responsible for the “turn” in the way of approaching emotions [22]; this could be a factor that explains, in our work, an adequate rethinking of individual functioning (occupational performance).

Regarding the phase of the disease, the scarcity of the sample (*n* = 19) may be one of the reasons that explains the lack of correlation in the mild phase of the disease and emotional intelligence, despite being the population group that showed higher levels of occupational performance. However, the moderate phase shows the relationship between emotional repair and self-care, while the severe phase reveals the relationship between emotional repair and leisure time. The skill demonstrates its benefits by covering a basic need (self-care) initially to give way to leisure in more advanced phases. The explanation can be found in the evolution of the coping process by the main caregiver. Based on the experience lived and reported by family members, the severe phase of the disease involves a stage of “relative discharge” as the behavioral and cognitive symptoms disappear, giving way to a state of “mental death”. Faced with this, the main caregiver is able to think more about themselves, without this implying the abandonment of the sick relative, allowing them the ability to find temporary relief. Furthermore, in this phase, the end of the patient’s life is anticipated and many caregivers report feeling a certain “relief” about the perceived burden that will decrease [47].

Our study has limitations that we can group from a theoretical and methodological point of view. Resilience is a concept that does not have, to date, a theoretical consensus, which makes an operational definition of the term difficult. As a result of this, confusion sometimes arises with other synonymous concepts, making it difficult to understand and evaluate.

Studies on resilience and emotional intelligence are proliferating in recent years, however, there is still a shortage in the study of certain populations, which makes it difficult to compare our work with others. Likewise, the fact of having wanted to study the joint influence of both variables prevents us, in a certain way, from comparing our results because we did not find previous studies that had the same objective.

It is also a limitation for the analysis of occupational performance not to have, to our knowledge, a standardized and validated assessment instrument for the Spanish population without disabilities that is clear, simple, with the self-report design, applicable in a reasonable time (similar to the rest of the attached scales, 30 min on average) and that does not require prior training that authorizes its administration.

For future research and intervention proposals derived from this study, we suggest carrying out a longitudinal design that allows for monitoring the evolution of the variables studied to facilitate and support the conclusion of results and develop interventions to train caregivers in emotional intelligence and resilience, and check if this improves the occupational performance studied in the present works. In addition, it would be interesting to know the degree of involvement of other family caregivers in the care of these patients, as their physical and psychological health may also be affected, especially if the COVID-19 pandemic situation may have influenced this care.

The fact that the survey was done through a self-reported questionnaire sampling method allowed us to reach a large sample. However, it also made it more difficult for the researchers to control the sample and important information for the research may have been lost. We consider that some questions about the use of any additional devices, the medical and surgical team involved in the treatments, or the surgery interventions received by the children could have been included in the questionnaire, making it more complete. Nevertheless, they were not included, as this would have meant a much larger questionnaire, increasing the risk of the participants not completing the surveys. 

Another possible limitation could have been the possible misunderstandings when completing the survey as the mother tongue of some participants was not English or Spanish, the languages in which our tool was designed. We believe that future research should take these considerations into account.

It should be noted that the use of self-reported questionnaires could introduce bias in the information received, as some questions may not have been clearly understood. However, the participants had the persons responsible for the study at their disposal to answer any questions and no queries were raised.

The study population consisted of main family caregivers of dementia patients who regularly participate in the different associations in the region of Extremadura. In total, the associations reported that this population consisted of 388 people, so the representativeness of the sample was 37.11%, which is considered a good participation rate, although it is always desirable to have a larger sample. We are sure that these results could be very interesting for our country as well as for other countries as they could help to know the benefit of training different skills in the face of certain stressful experiences in the care of these patients.

On the other hand, we did not find that the socio-demographic variables analyzed were related to the phases of the disease, which could indicate that these variables would have no effect on the results found. However, these results should be taken with caution due to the small size of some groups when dividing the sample according to severity stages and it would be of great interest to perform a regression analysis in future similar studies.

## 5. Conclusions

Based on the results obtained in our study, primary family caregivers with higher levels of resilience and emotional intelligence show better occupational performance. The stage of the patient’s disease could have an influence on this relationship.

## Figures and Tables

**Table 1:** Demographic outcomes by severity stage.

Sociodemographic Data				
		Percentage(n)		
		Mild	Moderate	Severe
Sex	Women	68.5(13)	70.5(62)	70.3(26)
	Men	31.6(6)	29.5(26)	29.7(11)
Level of Studies	Without studies	5.3(1)	17.0(15)	10.8(4)
	Basic Studies	63.2(12)	43.2(38)	56.8(21)
	Baccalaureate	26.3(5)	25.0(22)	13.5(5)
	Superior	5.3(1)	14.8(13)	18.9(7)
Age	20–30	5.3(1)	1.1(1)	0
	31–40	5.3(1)	2.3(2)	5.4(2)
	41–50	21.1(4)	20.5(18)	21.6(8)
	51–60	21.1(4)	25.0(22)	21.6(8)
	61 or more	47.4(9)	51.1(45)	51.4(19)
Relationship of Family Member With Dementia Patient	Mother	36.8(7)	47.7(42)	40.5(15)
	Spouse	36.8(7)	37.5(33)	43.2(16)
	Father	15.8(3)	10.2(9)	10.8(4)
	Father or Mother-in-law	5.3(1)	2.3(2)	0
	Brother or Sister	5.3(1)	1.1(1)	2.7(1)
	Grandma or Grandpa	0	1.1(1)	0
	Neighbor	0	0	2.7(1)

No relationship was found between the socio-demographic variables analyzed and the stage of the disease in which the patients were found. (sex: χ^2^ = 0.031; *p* = 0.984; level of education:χ^2^ = 7.049; *p* = 0.316; age:χ^2^ = 3.798; *p* = 0.875; family members:χ^2^ = 7.876; *p* = 0.795).

**Table 2:** Occupational performance questionnaire in caregivers.

Occupational Questionnaire		Percentage(n)	Satisfaction
Time dedicated to self-care	Not at all	4.2(6)	61.1% YES 38.9% NO
	Little	21.5(31)	
	Somewhat	29.9(43)	
	Quite a bit	31.9(46)	
	A lot	12.5(18)	
Time dedicated to productivity	Not at all	4.9(7)	65.3% YES 34.7% NO
	Little	4.2(6)	
	Somewhat	22.9(33)	
	Quite a bit	47.9(69)	
	A lot	20.1(29)	
Time dedicated to leisure activities	Not at all	20.8(30)	>32.6% YES 67.4% NO
	Little	37.5(54)	
	Somewhat	23.6(34)	
	Quite a bit	13.9(20)	
	A lot	4.2(6)	
Sleep		M ± SD	Restful Sleep
Hours of sleep per day		6.39 ± 1.36	40.6% YES 59.4% NO

Abbreviations: M, mean; SD, standard deviation.

**Table 3:** Mean scores of resilience, emotional intelligence, and occupational performance, global sample and according to phase of dementia.

Global Sample			Minimum–Maximum		M ± SD	
Emotional Intelligence			32–120		78.48 ± 4.82	
Resilience			28–97		64.01 ± 14.5	
Phases	Mild Phase		Moderate Phase		Severe Phase	
	Min–Max	M ± SD	Min–Max	M ± SD	Min–Max	M ± SD
Emotional Intelligence	64–120	84.47 ± 12.85	32–117	75.58 ± 15.58	49–102	79.00 ± 13.96
Resilience	28–96	69.37 ± 14.19	28–97	62.61 ± 15.51	34–83	62.53 ± 12.02

Abbreviations: M, mean; SD, standard deviation.

**Table 4:** Relationship between resilience and occupational performance in caregivers of patients with dementia. Global sample and differentiation according to the patient’s dementia stage.

Occupational Performance	Resilience CD-RISC 25	
	r *	*p*-Value
Global Sample (*N* = 144)		
Time dedicated to self-care	0.196	0.019
Time dedicated to productivity	0.091	0.278
Time dedicated to leisure activities	0.172	0.040
Dementia in mild phase (*N* = 19)		
Time dedicated to self-care	−0.036	0.882
Time dedicated to productivity	0.327	0.172
Time dedicated to leisure activities	−0.037	0.881
Dementia in moderate phase (*N* = 88)		
Time dedicated to self-care	0.227	0.033
Time dedicated to productivity	−0.045	0.679
Time dedicated to leisure activities	0.262	0.014
Dementia in severe phase (*N* = 37)		
Time dedicated to self-care	0.214	0.210
Time dedicated to productivity	0.355	0.034
Time dedicated to leisure activities	0.066	0.700

* Spearman’s Rho correlation coefficient.

**Table 5:** Relationship between emotional intelligence and occupational performance in caregivers of patients with dementia. Global sample and differentiation according to the patient’s dementia stage.

Occupational Performance	Emotional Intelligence TMMS-24	
	r *	*p*-Value
Global Sample (*N* = 144)		
Time dedicated to self-care	0.233	0.005
Time dedicated to productivity	0.056	0.508
Time dedicated to leisure activities	0.073	0.387
Dementia in mild phase (*N* = 19)		
Time dedicated to self-care	0.093	0.705
Time dedicated to productivity	0.125	0.611
Time dedicated to leisure activities	−0.095	0.698
Dementia in moderate phase (*N* = 88)		
Time dedicated to self-care	0.214	0.046
Time dedicated to productivity	0.026	0.807
Time dedicated to leisure activities	0.103	0.340
Dementia in severe phase (*N* = 37)		
Time dedicated to self-care	0.398	0.016
Time dedicated to productivity	0.130	0.449
Time dedicated to leisure activities	0.073	0.672

* Spearman’s Rho correlation coefficient.

**Table 6:** Relationship between the subscales of emotional intelligence and occupational performance in caregivers of patients with dementia. Global sample and differentiation according to the patient’s dementia stage.

Global Sample (*N* = 144)							
Emotional Intelligence		Occupational Performance					
		Self-Care		Productivity		Leisure Time	
		M ± SD	*p*-Value *	M ± SD	*p*-Value *	M ± SD	*p*-Value *
Attention	Should improve their attention; gives poor attention	3.16 ± 1.0	0.338	3.77 ± 1.0	0.553	2.30 ± 0.9	0.455
	Adequate attention	3.36 ± 1.1		3.75 ± 0.9		2.55 ± 1.2	
	Should improve their attention: gives too much attention	3.50 ± 0.9		3.60 ± 0.8		2.70 ± 1.5	
Clarity	Should improve their clarity	3.24 ± 1.1	0.951	3.67 ± 1.0	0.451	2.50 ± 1.1	0.404
	Adequate clarity	3.27 ± 1.1		3.74 ± 1.0		2.47 ± 1.1	
	Excellent clarity	3.38 ± 0.9		4.06 ± 0.8		2.13 ± 1.1	
Repair	Should improve their repair tactics	3.00 ± 0.9	0.075	3.87 ± 1.0	0.627	2.22 ± 1.0	0.019
	Adequate repair tactics	3.38 ± 1.2		3.68 ± 1.0		2.4 ± 1.1	
	Excellent repair tactics	3.50 ± 0.9		3.75 ± 0.9		3.19 ± 1.2	
Mild Dementia (*N* = 19)							
Attention	Should improve their attention; gives poor attention	3.50 ± 0.6	0.778	3.75 ± 0.5	0.752	2.75 ± 1.0	0.787
	Adequate attention	3.0 ± 1.2		3.69 ± 1.1		2.38 ± 1.0	
	Should improve their attention: gives too much attention	3.50 ± 0.7		3.50 ± 0.7		2.50 ± 0.7	
Clarity	Should improve their clarity	2.60 ± 1.5	0.203	3.40 ± 1.3	0.306	2.80 ± 0.8	0.554
	Adequate clarity	3.50 ± 0.7		3.67 ± 0.8		2.42 ± 0.9	
	Excellent clarity	2.50 ± 0.7		4.50 ± 0.7		2.00 ± 1.4	
Repair	Should improve their repair tactics	3.67 ± 0.6	0.614	3.67 ± 1.5	0.911	2.33 ± 0.6	0.727
	Adequate repair tactics	3.07 ± 1.1		3.67 ± 009		2.47 ± 1.0	
	Excellent repair tactics	3.00 **		4.00 **		3.00 **	
Moderate Dementia (*N* = 88)							
Attention	Should improve their attention; gives poor attention	3.24 ± 1.0	0.483	3.76 ± 1.1	0.924	2.37 ± 0.9	0.266
	Adequate attention	3.47 ± 1.1		3.79 ± 0.9		2.74 ± 1.2	
	Should improve their attention: gives too much attention	3.50 ± 1.3		3.75 ± 1.0		3.00 ± 1.6	
Clarity	Should improve their clarity	3.39 ± 1.0	0.825	3.64 ± 1.0	0.459	2.67 ± 1.1	0.639
	Adequate clarity	3.29 ± 1.2		3.88 ± 1.0		2.48 ± 1.1	
	Excellent clarity	3.50 ± 0.9		3.80 ± 0.6		2.50 ± 1.1	
Repair	Should improve their repair tactics	3.00 ± 0.9	0.029	3.94 ± 0.9	0.506	2.34 ± 1.1	0.305
	Adequate repair tactics	3.60 ± 1.2		3.62 ± 1.1		2.62 ± 1.0	
	Excellent repair tactics	3.36 ± 0.9		3.91 ± 0.7		2.91 ± 1.3	
Severe Dementia (*N* = 37)							
Attention	Should improve their attention; gives poor attention	2.89 ± 1.0	0.360	3.79 ± 1.1	0.721	2.05 ± 1.1	0.963
	Adequate attention	3.38 ± 1.3		3.69 ± 1.2		2.15 ± 1.2	
	Should improve their attention: gives too much attention	3.50 ± 1.0		3.50 ± 1.0		2.50 ± 1.2	
Clarity	Should improve their clarity	3.08 ± 1.0	0.806	3.85 ± 0.9	0.195	1.92 ± 1.0	0.138
	Adequate clarity	3.11 ± 1.2		3.47 ± 1.2		2.47 ± 1.3	
	Excellent clarity	3.50 ± 1.3		4.50 ± 1.0		1.25 ± 0.5	
Repair	Should improve their repair tactics	2.80 ± 0.9	0.184	3.70 ± 1.3	0.546	1.80 ± 0.6	0.014
	Adequate repair tactics	3.14 ± 1.2		3.82 ± 1.0		1.95 ± 1.2	
	Excellent repair tactics	4.00 ± 0.8		3.25 ± 1.3		4.00 ± 1.2	

* H is from Kruskal–Wallis, ** Unique case. It is not appropriate to calculate the standard deviation.

## Data Availability

The data underlying this article cannot be shared publicly to maintain the privacy of individuals that participated in the study. The data will be shared on reasonable request to the corresponding author.

## Supplementary Materials

The following are available online at https://www.mdpi.com/article/10.3390/jcm10184262/s1, Table S1: Demographic. psychosocial and clinical outcomes, Table S2: Psychosocial and clinical outcomes.
