# Supplementary material for: Resilience, Emotional Intelligence, and Occupational Performance in Family Members Who Are the Caretakers of Patients with Dementia in Spain: A Cross-Sectional, Analytical, and Descriptive Study
Source: J Clin Med. 2021 Sep 20;10(18):4262. doi: 10.3390/jcm10184262 (PMC8469665; doi:10.3390/jcm10184262)
Supplement: Supplementary file 1 [file jcm-10-04262-s001.zip › jcm-1320080-supplementary.pdf]

SUPPLEMENTAL MATERIAL

Table S1. Demographic. psychosocial and clinical outcomes.

SOCIODEMOGRAPHIC DATA

		% or/and M±SD (n)	
**SEX**	Women	70.1 % (101)	
	Men	29.9 % (43)	
**LEVEL OF STUDIES**	Without studies	13.9 %(20)	
	Basic Studies	49.3 %(71)	
	Baccalaureate	22.2 %(32)	
	Superior	14.60 %(21)	
**CIVIL_STATUS**	Married	79.2 %(105)	
	Living with partner	6.9 %(10)	
	Single	1.4 %(2)	
	Separated	12.5 %(18)	
**AGE**	20-30	1.4 %(2)	
	31-40	3.5 %(5)	
	41-50	28.8 %(41)	
	51-60	23.6 %(34)	
	61 or more	50.7 %(73)	
**Nº OF CHILDREN**	None	20.8 %(30)	
	1 child	5.6 %(8)	
	2 children	41 %(59)	
	3 children	22.2 %(32)	
	4 or more children	10.4%(15)	
**PEOPLE WHO LIVE AT HOME**		YES%	NO%
	Couple	79.9%(115)	20.1%(29)
	Children	37.5%(54)	62.5%(90)
	Mother	38.2%(55)	61.8%(89)
	Father	11.1%(16)	88.9%(128)
	Siblings	2.8%(4)	97.2%(140)
	Mother and Father-in-law	1.4 %(2)	98.6%(142)
	Caretakers	1.4 %(2)	98.6%(142)
	Grandchildren. grandparents. neighbor	2.8%(4)	97.2%(140)
		**2.93± 1.11**	
**EMPLOYMENT SITUATION**		YES%	NO%
	Work outside of the home/paid work	28.5%(41)	71.5 %(103)
**RELATIONSHIP OF FAMILY MEMBER WITH DEMENTIA PATIENT**	Mother	44%(64)	
	Spouse	38.9%(56)	
	Father	11.1%(16)	
	Father or Mother-in-law	2.1%(3)	
	Brother or Sister	2.1%(3)	
	Grandma or Grandpa	0.7 %(1)	

	Neighbor	0.7%(1)	

Abbreviations: M. mean; SD. standard deviation.

Table S2. Psychosocial and clinical outcomes

PSYCHOSOCIAL DATA			
**AGE OF THE PATIENT**	50-60 years	2.1 %(3)	
	61-70 years	10.4%(15)	
	71-80 years	41 %(59)	
	81-90 years	42.4 %(61)	
	90 or more years	4.2%(6)	
		YES%	NO%
**Type of Care**	Taking care of other people. besides the family member with dementia	37.5%(54)	62.5%(90)
	Hours of the day dedicated to the care of the sick family member	15.65± 7.71	
	Days of the week	6.86± 0.67	
	Time spent under care		
	< 6 months	1.4%(2)	
	6-12 months	9.2%(13)	
	1-2 years	14.8%(21)	
	2-4 years	29.6%(43)	
	4-6 years	14.1%(20)	
	>6 years	31.0%(45)	
		YES%	NO%
**SUPPORT**	**Formal support for the care of their family member**	85.4 %(123)	14.6 %(21)
	**Type of formal support**		
	Day Center	78 %(112)	
	Help at home	32.5%(47)	
	Major residencies	3.3%(5)	
	Economic presentation	8.9%(13)	
	Others	4.9%(7)	
	**Type of informal support**		
	Family	93.7%(135)	
	Friends	5.4%(9)	
		YES%	NO%
**LIVING THROUGH THE EXPERIENCE**	**Positive support**	72.2 %(104)	27.8 %(40)
	**Positive contributed feelings**		
	Love and surrender	77.9%(112)	

	Utility	37.5%(54)
	Gratitude toward the patient	29..8%(43)
	Personal Growth	38.5%(55)
	Others (empathy. respect)	2.9%(4)
	**Negative contributed feelings**	
	Overload	74.6%(107)
	Physical health problems	35.8%(55)
	Psychological health problems	35.8%(55)
	Others (embarrassment. worry. frustration)	4.4%(6)
	**CLINICAL DATA**	
**TYPE OF DEMENTIA**	Alzheimer	66.7 %(96)
	Vascular Dementia	13.2%(19)
	Lewy Body Dementia	2.8%(4)
	Frontotemporal Dementia	6.9%(10)
	Dementia associated with Parkinson's	4.2 %(6)
	Dementia mixture (Alzheimer + vascular)	4.9%(7)
	Other dementia types	1.4%(2)
**Dementia phase**	Mild	13.2 %(19)
	Moderate	61.1 %(88)
	Severe	25.7 %(37)

Abbreviations: M. mean; SD. standard deviation.
